# Sodium butyrate attenuates peritoneal fibroproliferative process in mice

**DOI:** 10.1113/EP090559

**Published:** 2022-12-02

**Authors:** Marcela Guimarães Takahashi De Lazari, Celso Tarso Rodrigues Viana, Luciana Xavier Pereira, Laura Alejandra Ariza Orellano, Henning Ulrich, Silvia Passos Andrade, Paula Peixoto Campos

**Affiliations:** ^1^ Department of General Pathology Institute of Biological Sciences Universidade Federal de Minas Gerais Belo Horizonte Minas Gerais Brazil; ^2^ Department of Biochemistry Institute of Chemistry University of São Paulo São Paulo São Paulo Brazil; ^3^ Department of Experimental Pathology Universidade Federal de São João del‐Rei Divinópolis Minas Gerais Brazil; ^4^ Department of Pathology University of Massachusetts Medical School Worcester Massachusetts USA; ^5^ Department of Physiology and Biophysics Institute of Biological Sciences Universidade Federal de Minas Gerais Belo Horizonte Minas Gerais Brazil

**Keywords:** angiogenesis, inflammation, remodelling, sponge implant

## Abstract

The aim of this study was to identify the bio‐efficacy of sodium butyrate (NaBu) on preventing the development of peritoneal fibrovascular tissue induced by implantation of a synthetic matrix in the abdominal cavity.

Polyether–polyurethane sponge discs were implanted in the peritoneal cavity of mice, which were treated daily with oral administration of NaBu (100 mg/kg). Control animals received water (100 μl). After 7 days, the implants were removed for assessment of inflammatory, angiogenic and fibrogenic markers.

Compared with control values, NaBu treatment decreased mast cell recruitment/activation, inflammatory enzyme activities, levels of pro‐inflammatory cytokines, and the proteins p65 and p50 of the nuclear factor‐κB pathway. Angiogenesis, as determined by haemoglobin content, vascular endothelial growth factor levels and the number of blood vessels in the implant, was reduced by the treatment. In NaBu‐treated animals, the predominant collagen present in the abdominal fibrovascular tissue was thin collagen, whereas in control implants it was thick collagen. Transforming growth factor‐β1 levels were also lower in implants of treated animals. Sodium butyrate downregulated the inflammatory, angiogenesis and fibrogenesis axes of the fibroproliferative tissue induced by the intraperitoneal synthetic matrix. This compound has potential to control/regulate chronic inflammation and adverse healing processes in the abdominal cavity.

## Abstract (highlights)

- **What is the central question of this study?**
  Peritoneal injury can result in a persistent fibroproliferative process in the abdominal cavity, causing pain and loss of function of internal organs. This study aimed to demonstrate the use of sodium butyrate (NaBu) as a potential agent to attenuate peritoneal fibrosis induced by a synthetic matrix.
- **What is the main finding and its importance?**
  Our findings provide the first evidence that NaBu attenuates the inflammatory, angiogenesis and fibrogenesis axes involved in the formation of peritoneal fibrovascular tissue, indicating the potential of this compound to ameliorate peritoneal fibrosis.

## 1 INTRODUCTION

Injury to the peritoneum induces a complex repair process that begins with the disruption of the mesothelial layer, resulting in brief vasoconstriction followed by increased vascular permeability and chemotaxis of inflammatory cells to the site of injury (Chegini, 2002; Mutsaers et al., 2015). In physiological healing, mesothelial cells stimulate fibrin deposition between 5 and 7 days, which is degraded in normal conditions after the release of fibrinolytic mediators from the mesothelial cells. In addition to mesothelial and polymorphonuclear cells, mast cells are also recruited/activated during peritoneal injury, releasing proteolytic enzymes that promote extracellular matrix deposition (Hu et al., 2020; Poerwosusanta et al., 2020; Yao et al., 2000). However, fibrinolytic imbalance causes subsequent deposition of extracellular matrix components by mesothelial cells, fibroblasts and myofibroblasts and persistent activation of the inflammatory and angiogenic cascades (Capella‐Monsonís et al., 2019; Cheong et al., 2001; Hellebrekers & Kooistra, 2011; Ozel et al., 2005). This results in the formation of fibrin bands between tissues and organs, which then become organized into fibrous tissue. The newly formed tissue is a dysmorphic connective structure composed of a dense, disorganized extracellular matrix, which is highly vascularized, differentiated, innervated, and populated with a variety of cell types. This aberrant healing process (fibrosis/abdominal adhesion) is responsible for impairing the functionality of visceral organs, constituting a major cause of morbidity and mortality (Capella‐Monsonís et al., 2019; Chegini, 2002; Gómez‐Gil et al., 2019; Ouaïssi et al., 2012).

Attenuation and/or inhibition of the components involved in this defective healing in the abdominal cavity would represent key targets not only to prevent this type of abdominal injury but also to stimulate physiological healing. In fact, anti‐inflammatory, anti‐angiogenic and anti‐fibrogenic compounds are now recognized to attenuate adhesions and other chronic inflammatory conditions in the peritoneal cavity (Capella‐Monsonís et al., 2019; Chegini, 2002). However, because of the complexity of the healing process in the peritoneal cavity, current interventions (physical and/or pharmacological) have failed to prevent/treat this pathological condition completely. This indicates that a search for new therapies is necessary (Atta, 2011; Capella‐Monsonís et al., 2019; Chegini, 2002).

Butyrate and other short‐chain fatty acids, derived from the gut microbiota, are major energy substrates for colonocytes and for the maintenance of intestinal homeostasis and functions (Canani et al., 2011). However, their activities extend to other organs and tissues beyond the intestines, exerting modulatory and integrative functions within biological systems. At the cellular level, the short‐chain fatty acids have been shown to modulate cell proliferation, differentiation, apoptosis and hormone secretion in both physiological and pathological processes (Hague et al., 1993; Kruh, 1981; Schwarz et al., 2017). In a number of systemic diseases, such as cancer, cystic fibrosis, obesity, ischaemic stroke, osteoarthritis and hypertrophic scars, where inflammation, angiogenesis and fibrogenesis co‐exist, butyrate and its derivatives have been shown to attenuate/modulate these events (Canani et al., 2011; Cleophas et al., 2019; Ghorbani et al., 2015; Pirozzi et al., 2018; Torii et al., 2017). For instance, sodium butyrate (NaBu) has been reported to reduce inflammatory cell recruitment/activation, to reduce mast cells and to inhibit the nuclear factor‐κB (NF‐κB) signalling pathway and production of pro‐inflammatory cytokines both in vitro and in vivo (Inatomi et al., 2005; Lee et al., 2017; Wang et al., 2018; Zhang et al., 2016). Our group also reported that oral administration of NaBu (de Lazari et al., 2020) was able to reduce inflammation induced by subcutaneous implantation of a synthetic matrix in mice (de Lazari et al., 2020). There is also evidence that NaBu induces or inhibits angiogenesis (Castro et al., 2020; Kim et al., 2007; Leek et al., 2012; Liu, Andrade, et al., 2016; Zgouras et al., 2003). These opposing effects are dependent on the dose, route of administration and experimental model (Liu, Andrade, et al., 2016). Sodium butyrate and its derivatives have also been shown to have an anti‐fibrogenic effect in human pterygium fibroblasts (Koga et al., 2017), to reduce the production of type I collagen in human lung fibroblasts (Rishikof et al., 2004), to inhibit the progression of chronic renal fibrosis (Wang et al., 2019) and to prevent liver dysfunction in a model of non‐alcoholic steatohepatitis (Ye et al., 2018). In a recent publication, Castro et al. (2020) have reported the modulatory effects of local application of low doses of NaBu on matrix remodelling during formation of subcutaneous granulation tissue induced by sponge implants (Castro et al., 2020).

Given that NaBu has been shown to target multiple pathways, such as those involved in physiological and pathological wound healing (inflammation, angiogenesis and fibrogenesis), and its oral bio‐efficacy, we reasoned that NaBu might modulate these processes after injury in the abdominal cavity. To test this hypothesis, we examined the effects of NaBu on our model of an implant‐induced abdominal wound. The tissue response to foreign body material implanted in the peritoneal cavity begins with an initial acute inflammatory phase. Within the first days (days 3–5), neutrophils and mast cells are activated and recruited to the site of the lesion, where a plethora of inflammatory mediators, signalling molecules and cytokines are released. A second influx of inflammatory cells, dominated by monocytes, is directed to the site, where they differentiate into macrophages that also produce/release an array of inflammatory molecules. At around days 5–7, formation of granulation tissue that follows the presence of macrophages is marked by the arrival of endothelial cells and fibroblasts. Angiogenesis occurs within the granulation tissue to supply blood flow to the inflamed environment. Activated fibroblasts are responsible for synthesis of extracellular matrix proteins and wound remodelling. In normal wound healing, inflammation and remodelling cease after repair. In contrast, in the foreign body response, the presence of the foreign material continues to induce inflammation, activates the formation of foreign body giant cells and leads to implant encapsulation by excessive deposition of collagen within and around the biomaterial (Anderson, 2000; Anderson & McNally, 2011; Kyriakides & Bornstein, 2003). In our model, we have observed a similar response to discs of polyether–polyurethane implanted in the peritoneal cavity of mice. The placement of a sponge implant intraperitoneally induces intense inflammatory and angiogenic responses followed by the formation of abdominal fibrovascular tissue inside and around the synthetic matrix. The newly formed tissue contains inflammatory cells, fibroblasts, neo‐vessels and soluble molecules (cytokines, growth factors and inflammatory mediators) embedded in the abundant extracellular matrix. In addition, this murine implant model has proved useful in assessing the effects of potential agents capable of modulating/controlling inflammatory processes and wound healing in the peritoneal cavity (Araújo et al., 2011; Marques et al., 2014; Mendes et al., 2007, 2009).

## 2 METHODS

### 2.1 Ethical approval

All animal procedures were approved by the Ethics Committee of Animal Use (CEUA) of the Universidade Federal de Minas Gerais (UFMG) under protocol number 282/2018. We selected 7‐ to 8‐week‐old male C57BL/6 mice (22–25 g body weight; *n* = 40) from the Animal Center (CEBIO). The animals were housed in individual cages after the surgical procedure and received food and water ad libitum. They were kept in a 12 h–12 h light–dark cycle. All experiments and postsurgical care were carried out according to the guidelines laid down by the animal welfare committee of the local institution and conformed to the principles and regulations, as described in the editorial by Grundy (2015).

### 2.2 Experimental design

Polyether–polyurethane sponges (Vitafoam, Manchester, UK) in disc format (5 mm thick × 8 mm in diameter) were used as implants to induce fibrovascular tissue growth in the intraperitoneal microenvironment. Before implantation, the sponges were soaked overnight in 70% v/v ethanol, then sterilized by boiling in distilled water for 30 min.

For the implant procedure, the animals were anaesthetized with a mixture of ketamine (150 mg/kg) and xylazine (10 mg/kg) (Syntec do Brasil LTDA, Brazil), administered intraperitoneally. The abdominal hair was shaved and the exposed skin wiped with 70% ethanol. The sterilized sponge discs were implanted inside the abdominal cavity through a 1‐cm‐long ventral mid‐line incision in the linea alba, which was closed with silk braided non‐absorbable suture material. The animals were monitored after surgery, and any signs of infection or discomfort were detected.

The animals were treated with sodium butyrate (Sigma; 100 mg/day/kg body weight; *n* = 20) diluted in filtered tap water (pH 7.4) and given in a final volume of 100 μl, which was administered by oral gavage daily for 6 days, according to our previous study and based on other experimental studies in animals (de Lazari et al., 2020; Lee et al., 2017). Control animals (*n* = 20) received 100 μl of filtered water. After 7 days, the implants were carefully dissected, weighed, and processed for multiple assays as described below. The animals were killed by cervical dislocation after sedation with an overdose (10 time more than the anaesthetic dose) of ketamine and xylazine.

### 2.3 Histological analysis

Post mortem, five animals in each group had the intraperitoneal implants dissected and fixed in 10% formalin for 24 h. After paraffin embedding, the sections (5 μm) were stained with Haematoxylin and Eosin (H s& E; for blood vessel counting) or Dominici (a mast cell marker) and processed for microscopic studies (Blue & Roberts, 1967). Picrosirius Red staining followed by polarized light microscopy was used to identify collagen deposition in the implants. To quantify the number of mast cells per field (area = 130.098 μm^2^ per field), images of 20 fields from histological cross‐sections from each implant were captured with a panchromatic objective lens (×40) in an optical microscope (final magnification ×400). To perform morphometric analysis of the number of blood vessels, images of cross‐sections obtained from 15 fields (area = 130.098 μm^2^ per field) were captured with a panchromatic objective lens (×40) of an optical microscope (final magnification ×400). A blood vessel was defined as a tube‐like structure with a lumen, whether or not it contained red blood cells. For analysis of collagen area, images from 40 fields of the cross‐section from each implant (area = 532.881 μm^2^ per field) were examined (×20 objective). The images were digitized and analysed using the software Image Pro‐Plus (Media Cybernetics, Rockville, MA, USA; Orellano et al., 2018). Processing and staining were performed by a technician who knew nothing about the samples or groups. At least two blinded investigators analysed the sections.

### 2.4 Activity of myeloperoxidase and *N*‐acetyl‐β‐d‐glucosaminidase within the implant

Neutrophil invasion into implants was assessed indirectly, by quantifying myeloperoxidase (MPO) activity, as previously described (Araújo et al., 2011; de Lazari et al., 2020; Marques et al., 2014; Mendes et al., 2007, 2009). Implant supernatants were weighed, homogenized in pH 4.7 buffer (0.1 M NaCl, 0.02 M NaPO_4_ and 0.015 M NaEDTA), and centrifuged for 10 min at 12,000*g*. The pellets were then resuspended in 0.05 M NaPO_4_ buffer (pH 5.4) containing 0.5% hexadecyltrimethylammonium bromide (HTAB), followed by three freeze–thaw cycles using liquid nitrogen. The MPO activity in the supernatant samples was assayed by measuring the change in absorbance (optical density; OD) at 450 nm using tetramethylbenzidine (1.6 mM) and H_2_O_2_ (0.3 mM). The reaction was terminated by the addition of 50 ml of H_2_SO_4_ (4 M). Results are expressed as a change in OD per gram of wet tissue.

The activity of the lysosomal enzyme *N*‐acetyl‐β‐d‐glucosaminidase (NAG; Araújo et al., 2011; de Lazari et al., 2020; Mendes et al., 2007, 2009) was determined in the implants by homogenizing the samples in NaCl solution (0.9% w/v) containing 0.1% v/v Triton X‐100 (Promega, Madison, WI, USA) and centrifugation (3,000*g*; 10 min at 4°C). One hundred microlitres of the supernatant was incubated for 10 min with 100 μl of *p*‐nitrophenyl‐*N*‐acetyl‐β‐d‐glucosaminide (Sigma‐Aldrich, St. Louis, MO, USA) prepared in citrate/phosphate buffer (0.1 M citric acid and 0.1 M Na_2_HPO_4_; pH 4.5) at a final concentration of 2.24 mM. The reaction was stopped by the addition of 100 μl of 0.2 M glycine buffer (pH 10.6). Hydrolysis of the substrate was determined by measuring the absorption at 400 nm. Results were expressed as nanomoles per milligram of wet tissue.

### 2.5 Measurement of cytokine levels in sponge implants

Production of the cytokines C‐X‐C motif chemokine ligand 1 (CXCL1), C‐C motif chemokine ligand 2 (CCL2), tumor necrosis factor alpha (TNF‐α) and transforming growth factor‐β1 (TGF‐β1) and vascular endothelial growth factor (VEGF) was determined by immunoassay kits (R&D Systems, USA) according to the manufacturer's protocol. The implants were homogenized in PBS (pH 7.4) containing 0.05% Tween and centrifuged for 30 min at 10,000*g*. Supernatants (50 μl) were added in duplicate to enzyme‐linked immunosorbent assay plates coated with a specific murine monoclonal antibody against the cytokine, followed by the addition of a second horseradish peroxidase‐conjugated polyclonal antibody, also against the cytokine (de Lazari et al., 2020). After washing to remove any unbound antibody–enzyme reagent, a substrate solution (50 μl of a 1:1 solution of hydrogen peroxide and 10 mg/ml tetramethylbenzidine in DMSO) was added to the wells. Colour development was halted after 20 min incubation with 2 N sulfuric acid (50 μl), and the intensity of the colour was measured at 540 nm on a spectrophotometer (Thermo Fisher Scientific). Standards were 0.5‐log_10_ dilutions of recombinant murine cytokines from 7.5 to 1,000 pg/ml (100 μl). The results were expressed as picograms of cytokine per milligram of wet tissue (de Lazari et al., 2020).

### 2.6 Western blot

Intraperitoneal implants from both groups (*n* = 5 in each group) were immediately frozen in liquid nitrogen after removal. The samples were prepared according to the protocol described by Baldwin (1996) and modified by de Lazari et al. (2020). The frozen samples were homogenized with the addition of lysis buffer (150 mM NaCl, 50 mM Tris–HCl, 5 mM EDTA‐2Na and 1 mM MgCl_2_) containing 1% nonidet P40, 0.3% Triton X‐100 and 0.5% SDS and a cocktail of protease inhibitors (SigmaFAST; Sigma) and phosphatase inhibitors (20 mmol/L NaF and 0.1 mmol/L Na_3_VO_4_) (Sigma). The cytoplasmic extract was removed to a clean tube. Nuclear extract (NE) buffer [1× solution composed of 20 mM Tris–Cl, 420 mM NaCl, 1.5 mM MgCl_2_, 0.2 mM EDTA, 1 mM PMSF and 25% glycerol (v/v), pH 8.0] was added to the nuclear pellet (50 μl). The salt concentration was adjusted to 400 mM using 5 M NaCl (by adding ∼35 μl), and the solution was completed with NE buffer (50 μl). The pellet was resuspended and incubated on ice for a period of 10 min; then 50 μg of protein was denatured and separated in denaturing SDS/7.5% polyacrylamide gel. The proteins were transferred to a nitrocellulose membrane (Merk Millipore, Burlington, MA, USA). The blots were blocked at room temperature with 2.5% skimmed milk powder in PBS plus 0.1% Tween 20 (de Lazari et al., 2020).

We used Tween 20 before incubation with polyclonal rabbit anti‐p65 (SC372; 1:1,000), mouse monoclonal anti‐p50 (SC166588; 1:500) and mouse monoclonal anti‐β‐tubulin (SC8035; 1:1,000) overnight, in a refrigerated room. The test showed immunoreactive bands using fluorescence collectors purchased from Santa Cruz Biotechnology (Santa Cruz, CA, USA) and using the Typhoon FLA 9000 scanner (GE Healthcare, Sweden). For densitometry analysis, the software NIH ImageJ was used (de Lazari et al., 2020).

### 2.7 Haemoglobin extraction

The amount of haemoglobin (Hb) was measured using the Drabkin method (Drabkin, 1946). The implants were homogenized individually in 1 ml of Drabkin's reagent (Labtest, São Paulo, Brazil) and centrifuged at 10,000 *g* for 20 min. The supernatants were filtered (0.22 μm filter; Millipore, São Paulo, Brazil), and the Hb concentration was determined spectrophotometrically by measuring absorbance at 540 nm and compared against a standard Hb curve. The content of Hb in the implant sponge was expressed as micrograms of Hb per milligram of wet tissue (Araújo et al., 2011; de Lazari et al., 2020; Marques et al., 2014; Mendes et al., 2007, 2009).

### 2.8 Soluble collagen measurement

Total soluble collagen was measured in whole tissue homogenates by the Picrosirius Red reagent‐based assay (Marques et al., 2014; Mendes et al., 2009). The implants were homogenized in 1 ml of PBS, and 50 μl of the tissue homogenate obtained was mixed with 50 μl of the reagent. The collagen–dye complex was precipitated by centrifugation for 10 min at 5,000*g*. The supernatant was drained off, and the pellet was washed with 500 μl of ethanol (99% pure and methanol free). One millilitre of a 0.5 M NaOH solution was added to the pellet of collagen‐bound dye. After solubilization, samples were transferred to a 96‐well plate and read at 540 nm. A calibration curve was set up based on a gelatin standard (Merck). The results are expressed as micrograms of collagen per milligram of wet tissue.

### 2.9 Statistical analysis

The assumptions of normality and homoscedasticity were determined for subsequent statistical analysis. All data were expressed as the mean ± SEM. Comparisons between the groups were made using Student's unpaired *t* test. Differences between means were considered significant when *P*‐values were <0.05. Statistical analysis was performed using the program GraphPad Prism, v.6.0 (Dotmatics, California, USA).

## 3 RESULTS

Here, we tested the capability of NaBu to prevent the development of peritoneal fibrovascular tissue induced by the implantation of a synthetic matrix in the abdominal cavity. The treatment was safe, in that oral administration of NaBu (100 mg/kg, for 7 days consecutively) did not result in any apparent signs of toxicity in the mice, such as sedation, weight loss or changes in motor activity. No infection or rejection was observed in the implant location during the 7 day period of the experiment.

**FIGURE 1:**
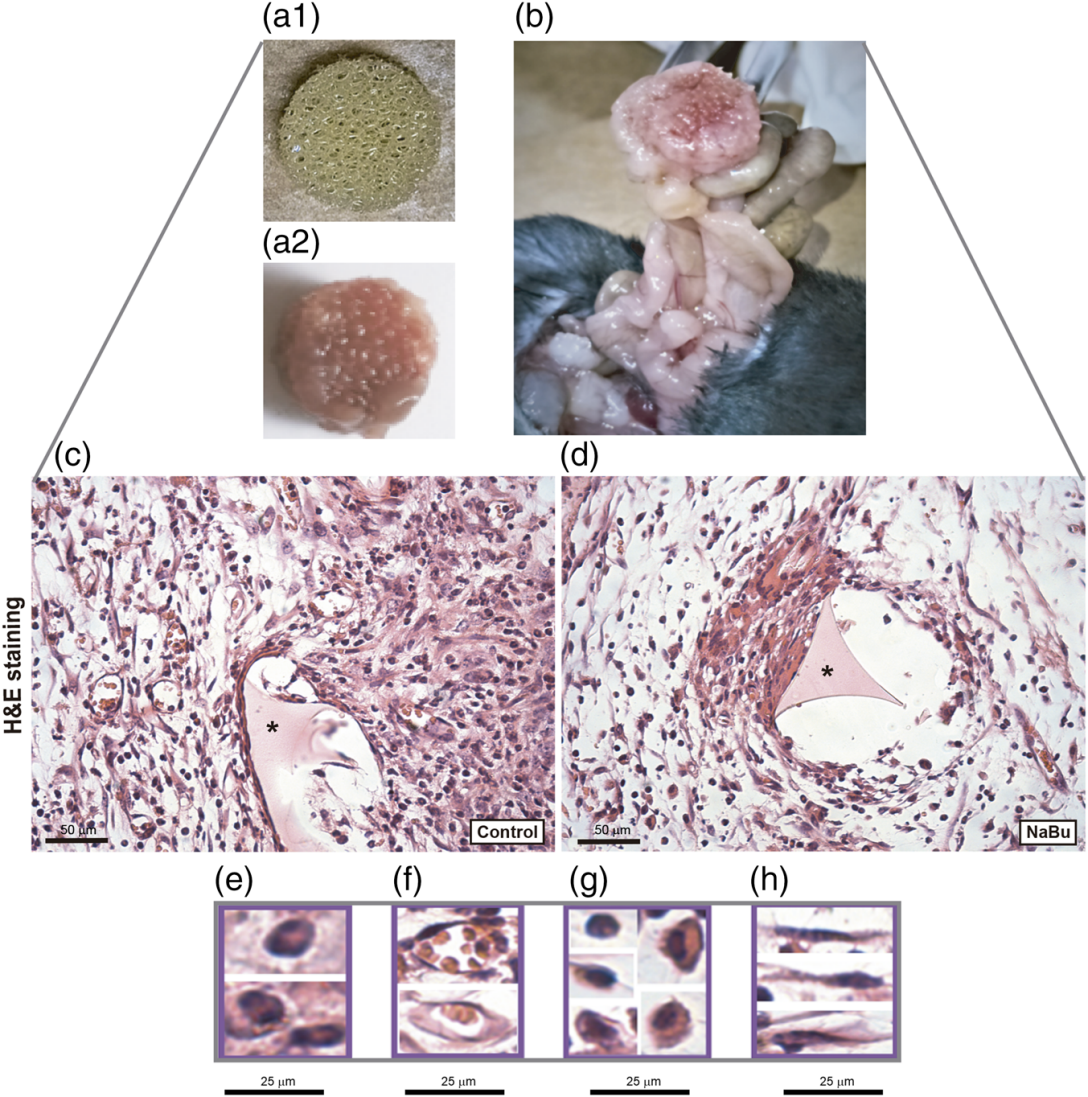
In situ image of polyether–polyurethane intraperitoneal implant and histological images of the fibrovascular tissue before and after sodium butyrate (NaBu) administration. (a,b) Representative images of the sponge disc before (a1) and after implantation in the peritoneal cavity (b) and of an excised implant after removal from the peritoneal cavity (a2). At 7 days post‐implantation, the synthetic matrix in situ is adhered to the intestine and liver by fibrous tissue. (c,d) Representative histological sections of intraperitoneal implants stained with Haematoxylin and Eosin (H & E) are shown. Abundant fibrovascular tissue and a greater number of vessels are seen in implants of control mice compared with the implants of treated animals. (e–h) Different cell types were identified morphologically: polymorphonuclear cells (e), endothelial cells (f), mononuclear cells (g) and spindle cells (h). *Sponge matrix

**FIGURE 2:**
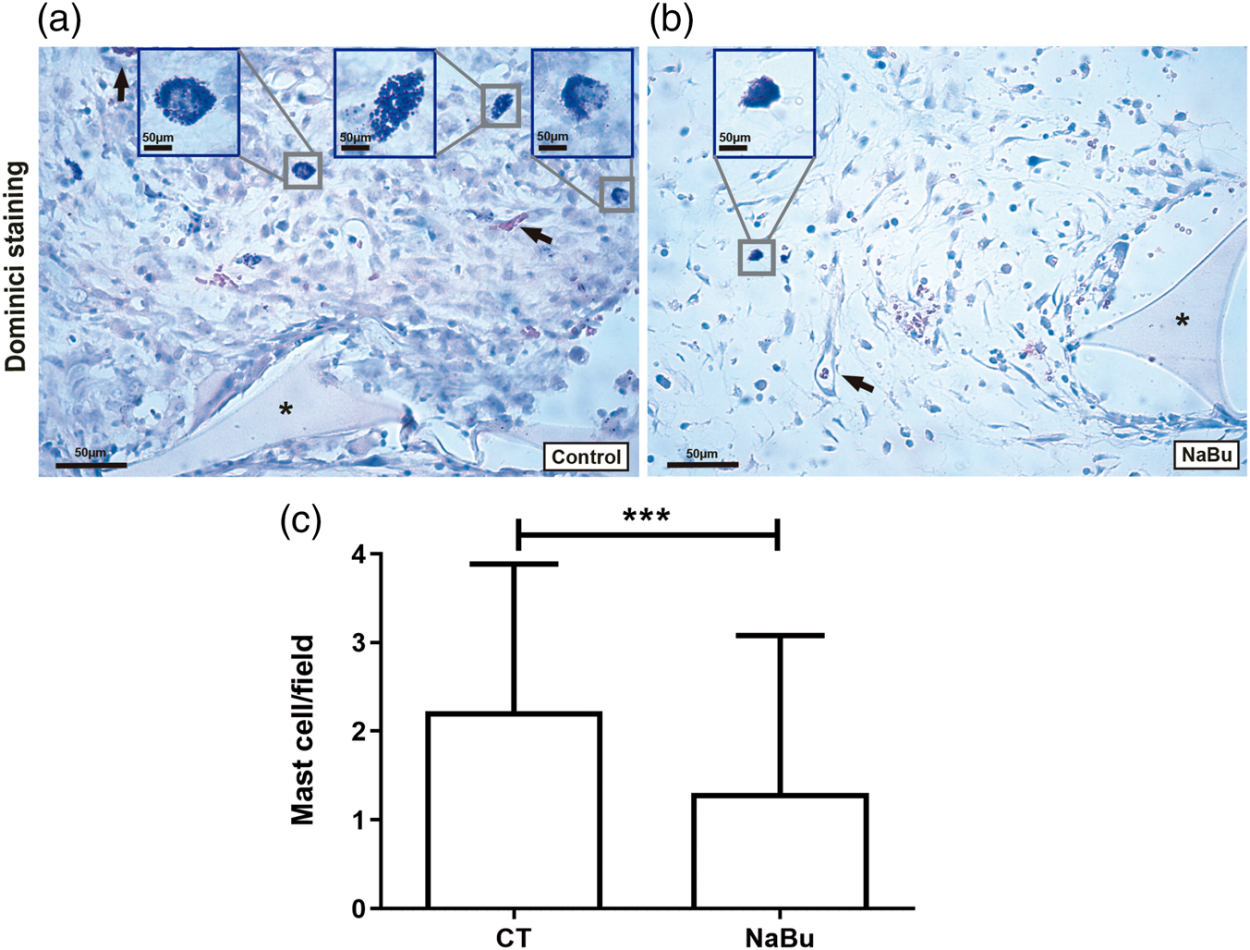
Effects of oral administration of sodium butyrate (NaBu) on the number of mast cells in intraperitoneal implants (*n* = 5 animals). (a,b) Representative histological sections of intraperitoneal implants stained with Dominici. (c) The number of mast cells is clearly decreased in implants of NaBu‐treated animals compared with the untreated group (CT). Values are means ± SD of five animals in each group. ^***^ *P* < 0.001, Student's unpaired *t* test. *Sponge matrix. Arrows indicate blood vessels

Implantation of polyether–polyurethane matrix induced a fibrovascular tissue that adhered to part of the intestine (adhesion‐like tissue; Figure 1a,b). The tissue that formed in animals administered with NaBu was less dense than that in control animals. Histological analysis of control implants and implants of NaBu‐treated mice stained with H&E revealed inflammatory cells, spindle‐shaped fibroblast‐like cells, blood vessels and extracellular matrix fibres in the fibrovascular tissue formed inside and around the sponge matrix (Figure 1c,d). However, extracellular matrix deposition and inflammatory cells were decreased in the implants of treated animals compared with the untreated group. The number of mast cells, detected in implants stained with Dominici, was lower in implants of NaBu‐treated mice compared with the control group (number of mast cells in control group 3.0 ± 0.3 vs. NaBu‐treated group 1.0 ± 0.4; Figure 2).

**FIGURE 3:**
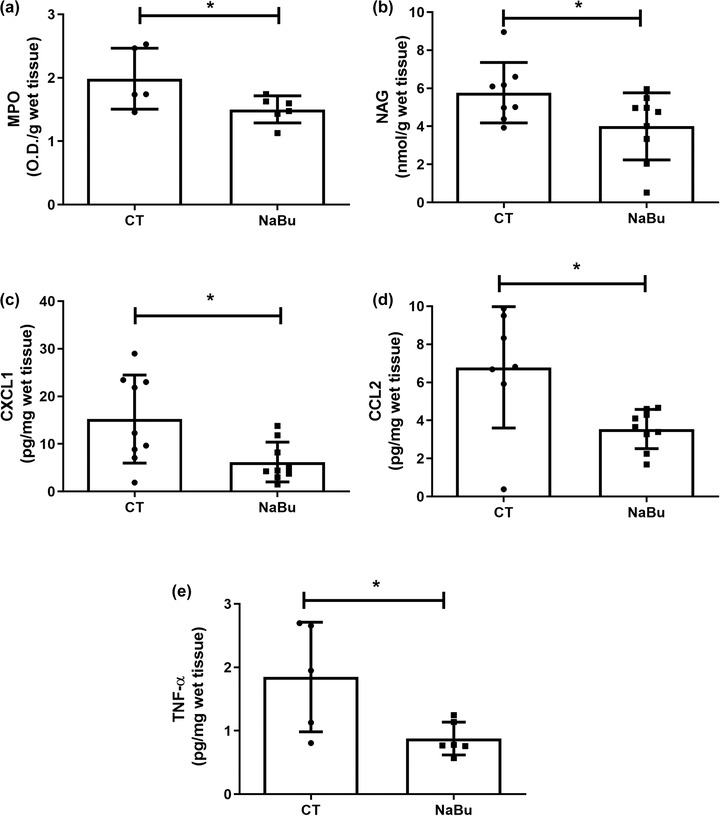
Effects of oral administration of sodium butyrate (NaBu) on inflammation induced by intraperitoneal implants. The expression of all inflammatory markers [myeloperoxidase (MPO; a); *N*‐acetyl‐β‐d‐glucosaminidase (NAG; b), C‐X‐C motif chemokine ligand 1 (CXCL1; c), C‐C motif chemokine ligand 2 (CCL2; d) and tumor necrosis factor alpha (TNF‐α; e)] decreased after the treatment. Abbreviation: CT, control group. Values are means ± SD of 6–10 animals in each group. ^*^ *P* < 0.05, Student's unpaired *t* test

**FIGURE 4:**
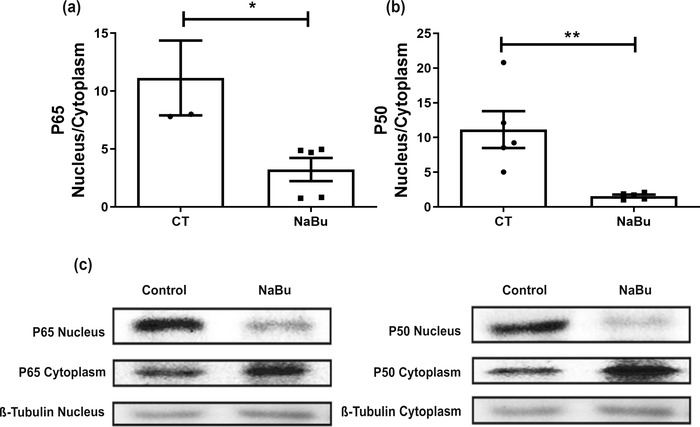
Effects of oral administration of sodium butyrate (NaBu) on activation of the transcription factor nuclear factor‐κB (NF‐κB) in intraperitoneal implants. (a,b) Sodium butyrate decreased the activation of this transcription factor, as shown by the relationship of nuclear to cytoplasmic p65 (a) and p50 (b). (c) Representative images of the blots are shown. Abbreviation: CT, control group. Values are the means ± SD of five animals in each group. ^*^ *P* < 0.05 and ^**^ *P* < 0.01, Student's unpaired *t* test

**FIGURE 5:**
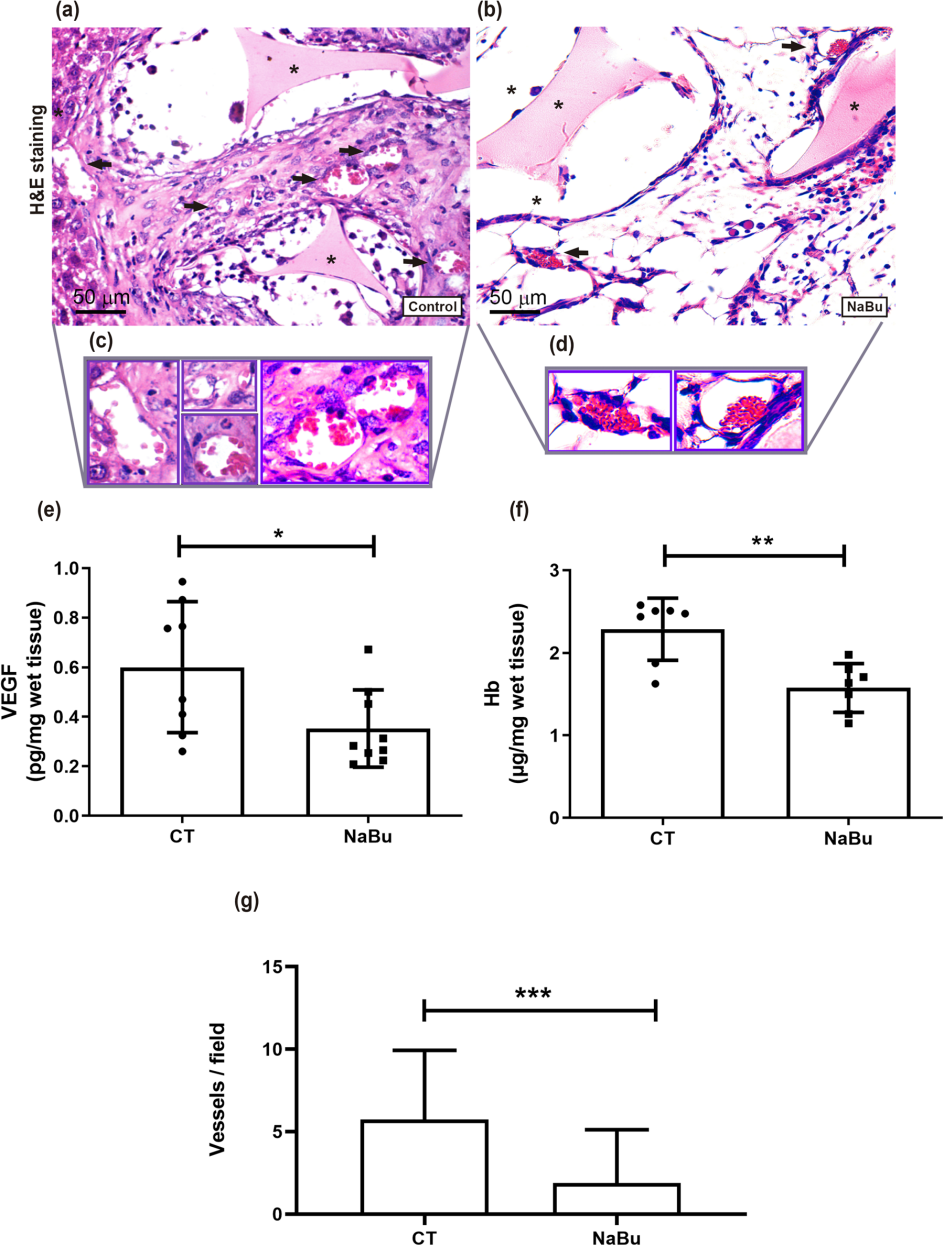
Effects of oral administration of sodium butyrate (NaBu) on angiogenesis induced by intraperitoneal implants. (a–d) Representative histological sections of intraperitoneal implants stained with Haematoxylin and Eosin (H & E; a, control implant; b, implant from an NaBu‐treated animal; c,d, vessels of different diameters from both implants are shown). (e,f) Vascular endothelial growth factor (VEGF) levels (e) and haemoglobin (Hb) content (f) decreased after NaBu treatment. (g) Morphometric analysis showed a reduction in the number of blood vessels in implants of NaBu‐treated group compared with the control (CT) implant. Values are means ± SD of five to nine animals in each group (*n* = 9 for Hb and VEGF; *n* = 5 for histological analysis). ^*^ *P* < 0.05, ^**^ *P* < 0.01 and ^***^ *P* < 0.001, Student's unpaired *t* test

We assessed the inflammatory response in the implants by quantifying the activity of enzymes involved in inflammation, the production of cytokines and activation of the transcription factor NF‐κB (Figures 3 and 4). All inflammatory markers examined were lower in implants of NaBu‐treated animals compared with the control group. The treatment reduced production of the inflammatory cytokines TNF‐α, CXCL1 and CCL2 by ∼50%. Implants of NaBu‐treated animals showed a significant reduction in the nuclear‐to‐cytoplasmic ratio of the NF‐κB heterodimer, which consists of the proteins p65 and p50 (Figure 4), as revealed by Western blot analysis. Likewise, NaBu reduced the levels of vascular endothelial growth factor (VEGF), haemoglobin content and the number of blood vessels, suggesting diminished angiogenesis (Figure 5). In control implants, VEGF levels were 0.60 ± 0.09 pg/mg, versus 0.35 ± 0.052 pg/mg after NaBu treatment. Haemoglobin content decreased from the control value of 2.28 ± 0.14 μg/mg wet tissue to 1.57 ± 0.11 μg/mg wet tissue after NaBu administration. These results were corroborated by the decreased number of blood vessels, examined in histological analysis of implant sections stained with H&E. The number of blood vessels in the control group was 6.0 ± 0.2, versus 2.0 ± 0.1 in the treated group (Figure 5g).

**FIGURE 6:**
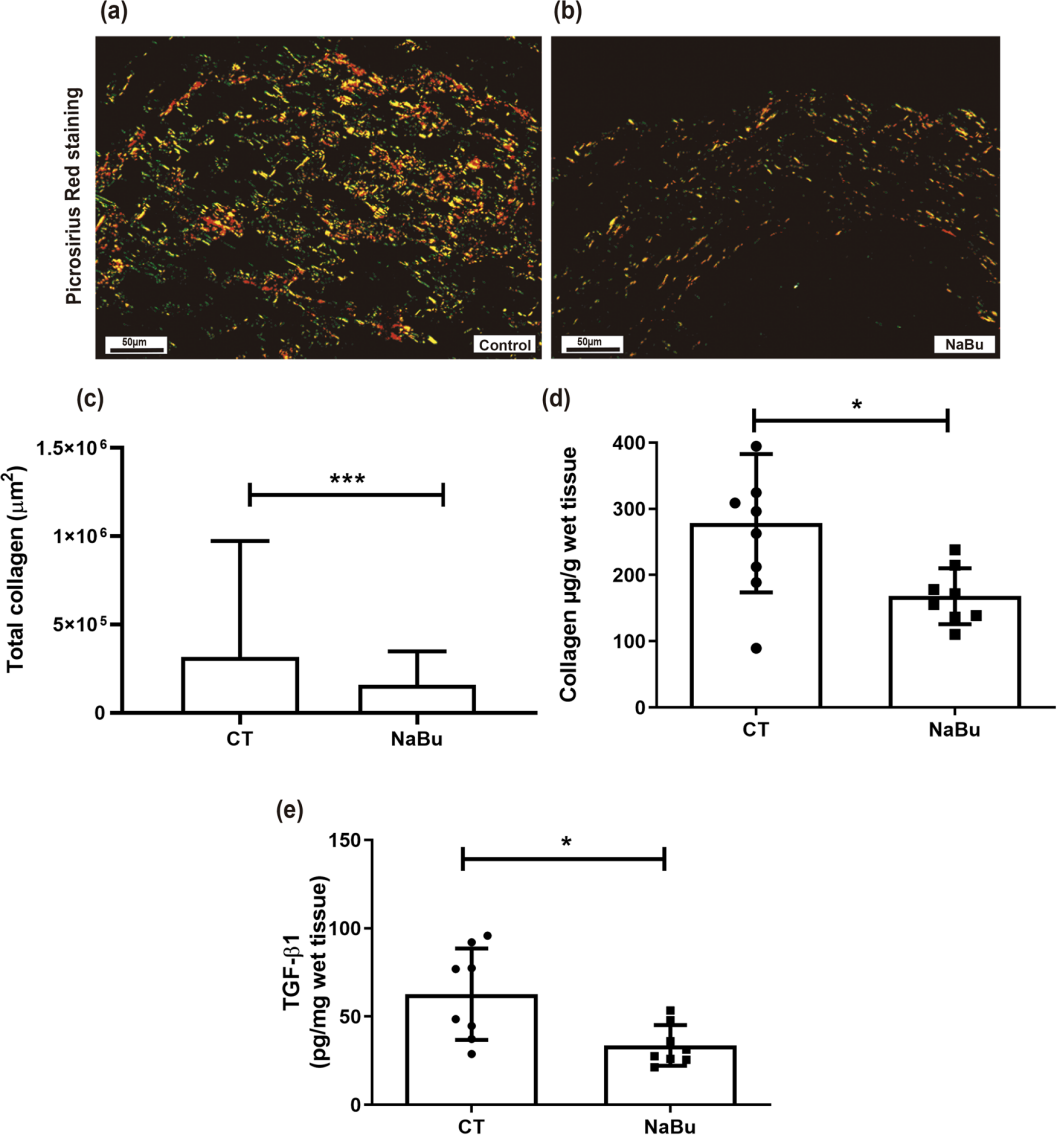
Effects of oral administration of sodium butyrate (NaBu) on markers of fibrogenesis induced by intraperitoneal implants. (a,b) Representative histological sections of intraperitoneal implants stained with Picrosirius Red (a, control implant; b, implant from an NaBu‐treated animal) show distinct types of collagen in the implants: type 1 (predominance of reddish colour) and type 3 (predominance of greenish colour). (c) Morphometric analysis shows that the treatment decreased the amount of collagen deposition in implants of NaBu‐treated animals compared with control (CT) implants. (d) Effects of NaBu on total soluble collagen. (e) Results of measurement of the pro‐fibrogenic cytokine transforming growth factor‐β1 (TGF‐β1) in implants from control and NaBu‐treated animals. Values are means ± SD of five to nine animals in each group (*n* = 5 for Picrosirius Red; *n* = 9 for soluble collagen and TGF‐β1). ^*^ *P* < 0.05 and ^***^ *P* < 0.001, Student unpaired *t* test

Sodium butyrate treatment attenuated fibrosis, as demonstrated by the decrease in the total area of collagen deposition in the implants, evaluated by Picrosirius Red staining and by the reduction in total soluble collagen in the treated group. In implants of NaBu‐treated animals, the collagen fibres were thinner and sparse, and the predominant type was type 3. In contrast, in control implants, increased collagen deposition was observed, and the predominant type was collagen type 1 (Figure 6a–d). A significant reduction in TGF‐β1 levels (50%) within the implant was observed in the treated group compared with the levels in control implants (Figure 6e).

## 4 DISCUSSION

Abdominal wound healing that occurs after injury is a multifactorial physiological process that involves overlapping events such as inflammation, angiogenesis and fibrogenesis. An imbalance between these processes, which involve different cell types, their interactions and complex molecular mechanisms, can result in defective peritoneal healing, such as adhesion and fibrosis, which are serious medical conditions and major challenges for the health system (Capella‐Monsonís et al., 2019; Chegini, 2002).

The data presented here demonstrate that oral administration of NaBu (100 mg/kg), a synthetic form of short‐chain fatty acid, attenuated the inflammatory, angiogenic and fibrogenic components of implant‐induced peritoneal fibrovascular tissue in mice. In the newly formed fibrovascular tissue inside and around the implant matrix, inflammatory cells, cytokine production, blood vessel formation and extracellular matrix deposition were identified. Similar features are found in healing processes in different tissues in both human and experimental animal models.

Histological analysis showed decreased inflammatory recruitment of immune cells, including mast cells, in addition to reduced numbers of blood vessels and reduced extracellular matrix deposition in implants of treated animals compared with the untreated group. Previous studies have shown that systemic administration of NaBu reduced neutrophil recruitment in a murine colitis model. Another study showed that oral NaBu supplementation decreased mast cell activation and the production of inflammatory mediators in a model of weaned pigs (Wang et al., 2018). Production of the inflammation markers MPO, NAG, CXCL1, CCL2, TNF‐α and the transcription factor NF‐κB decreased in implants of Nabu‐treated animals compared with those in the untreated group. Activities of enzymes associated with inflammation, such as MPO and NAG, are well‐established assays to determine the numbers of neutrophils and macrophages indirectly in inflammatory sites and processes, including those in the sponge implant (Araújo et al., 2011; de Lazari et al., 2020; Marques et al., 2014; Mendes et al., 2007, 2009). Myeloperoxidase activity, for example, was used as an inflammatory parameter to evaluate the effects of butyrate in a rat colitis model (Butzner et al., 1996; Simeoli et al., 2017). The other soluble inflammatory markers used in this work have been shown to be involved in a number of acute and chronic inflammatory processes, acting in the recruitment/activation of inflammatory cells (Hayden & Ghosh, 2012; Kany et al., 2019; Leppkes et al., 2014). Our results are in agreement with several publications that have shown the anti‐inflammatory effects of butyrate and/or its derivatives through inhibition of cytokine production and attenuation of the phosphorylation of NF‐κB. In a model of experimental colitis, oral administration of butyrate has been shown to decrease TNF‐α, to block NF‐κB signalling and to reverse histone acetylation (Lee et al., 2017). Butyrate was shown to protect the gastric mucosa against ethanol‐induced lesions by decreasing both parameters (Liu, Wang, et al., 2016). A reduction in CCL2 and TNF‐α production was observed after oral administration of a butyrate derivative in mice with colitis (Simeoli et al., 2017). In our model of inflammation induced by subcutaneous implantation of synthetic biomaterial, we have shown that sodium butyrate was able to decrease cytokine production and activation of NF‐κB (de Lazari et al., 2020). However, to our knowledge, there are no reports on the effects of NaBu on the inflammatory component of abdominal wound processes. Thus, our work is the first demonstration of such an effect.

We have also examined the effects of NaBu on angiogenesis (numbers of blood vessels, VEGF levels and Hb levels) in the abdominal implant. Our findings showed that these parameters were decreased by the treatment. This inhibitory response is likely to involve the effects of histone deacetylase inhibition exerted by NaBu (Falkenberg & Johnstone, 2014). However, some reports have shown that NaBu has either increased or decreased angiogenesis in vivo and in vitro. This variation is dependent on the dose, route of administration and experimental model used (Castro et al., 2020; de Lazari et al., 2020; Kim et al., 2007).

It has been reported that inflammation and angiogenesis are crucial factors for the formation of fibrovascular tissue in physiological and pathological healing processes (Atta, 2011; Capella‐Monsonís et al., 2019). In fact, we have also found that in addition to inhibiting inflammation and angiogenesis, NaBu attenuated key parameters of fibrogenesis (collagen deposition and TGF‐β levels) in intraperitoneal implants. The treatment influenced not only the amount of collagen deposition, but also the pattern and type of collagen. More mature collagen (type 1) was observed in implants of control animals compared with that of treated mice (type 3, immature collagen). These results are important because, to our knowledge, there are no previous reports on the effects of NaBu on the pattern of collagen deposition in healing processes. Our findings are in agreement with several reports that have shown the anti‐fibrogenic effects of NaBu in in vitro and in vivo systems by downregulating the expression, production and deposition of fibrotic markers (Koga et al., 2017; Rishikof et al., 2004; Wang et al., 2019; Ye et al., 2018). Transforming growth factor‐β is recognized to regulate both physiological and pathological wound repair (Chegini, 2008); therefore, it is possible that NaBu also targeted the fibrogenic cascade involved in extracellular matrix deposition in our model through the downregulation of TGF‐β. In a recent report, it was shown that a low concentration of NaBu applied directly within the implant was able directly to activate and stimulate wound‐healing properties of fibroblasts, promoting matrix remodelling and maturation (Castro et al., 2020). These contrasting results might be attributed to the different dose/route of administration used, thus low doses of NaBu injected at the site of the injury appear to be pro‐fibrogenic, whereas the dose and/or route of administration used in this work exerted an anti‐fibrogenic effect.

Studies on bioavailability after oral administration of NaBu (100 mg/kg) showed an increased butyric acid concentration in mouse serum 20 min after ingestion of the compound (Russo et al., 2021). Whether the effects of NaBu on the peritoneal wound parameters were obtained through direct/indirect actions of the butyric acid remains to be determined.

In our study, the injury caused by the presence of polyether–polyurethane implants in the peritoneal cavity resulted in the formation of abdominal fibrovascular proliferating tissue. Oral administration of NaBu showed bio‐efficacy by downregulating the main axes (inflammation, angiogenesis and fibrogenesis) during the process. Our findings extend the range of actions of this short fatty acid as a potential systemic treatment to control/regulate chronic inflammation and tissue repair after abdominal injury, thus suggesting a relevant regulatory role of this endogenous biomolecule in other sites beyond the intestines.

## AUTHOR CONTRIBUTIONS

Marcela G. T. De Lazari, Celso T. R. Viana, Luciana X. Pereira and Laura A. A. Orellano conducted the experimental study, data acquisition, analysis and manuscript preparation. Paula P. Campos and Henning Ulrich conducted the manuscript editing, literature research and data analysis. Silvia P. Andrade conducted study concept and design, manuscript preparation and review. All authors approved the final version of the manuscript and agree to be accountable for all aspects of the work in ensuring that questions related to the accuracy or integrity of any part of the work are appropriately investigated and resolved. All persons designated as authors qualify for authorship, and all those who qualify for authorship are listed.

## CONFLICT OF INTEREST

None declared.

## Supporting information

Statistical Summary Document

## Data Availability

The authors confirm that the data supporting the findings of this study are available within the article and its Supporting Information. The data that support the findings of this study are available from the corresponding author upon reasonable request.
